# In vivo comparison of DENSE and CSPAMM for cardiac motion analysis

**DOI:** 10.1186/1532-429X-11-S1-P73

**Published:** 2009-01-28

**Authors:** Christian T Stoeck, Sebastian Kozerke, Neil Maredia, Andrew Crean, John P Greenwood, Sven Plein

**Affiliations:** 1 Institute for Biomedical Engineering, University and ETH, Zurich, Switzerland; 2 Academic Unit of Cardiovascular Medicine, University of Leeds, Leeds, UK

**Keywords:** Cardiac Motion, Line Distance, Phase Unwrap, Hold Position, Fourth Order Polynomial

## Introduction

Tagging has shown great promise for analyzing cardiac motion patterns [1]. Two different methods have been proposed to utilize the phase associated with harmonic modulation of magnetization to enable tissue tracking. Using Displacement ENcoding with Stimulated Echoes (DENSE) [2] tagged magnetization is demodulated by decoding gradients permitting motion tracking from the signal phase. In contrast, HARmonic Phase (HARP) [3] analysis of Complementary SPAtial Modulation of Magnetization (CSPAMM) data decomposes tagged data into its harmonic components during post-processing. It has been argued in principle that the information content of DENSE and HARP should be identical [4]. However, a formal comparison of DENSE and HARP has not been undertaken to date. In this study, DENSE and HARP data were obtained consecutively in the same subjects and circumferential shortening, rotation and time to peak motion were evaluated.

## Methods

Eight healthy volunteers were imaged using 2D CSPAMM [5] and 2D DENSE [6] with identical scan duration (~14 sec). Data were read out using an EPI sequence with the following parameters: TR/TE/α = 30 ms/5.3 ms/20°, acquisition matrix of 96ξ42 (CSPAMM) or 48ξ40 (DENSE) reconstructed to 192ξ192, FOV of 320 mmξ253 mm, slice thickness of 8 mm. Tagging was applied using 2× lines (line distance: 8 mm) employing two orthogonal imaging stacks.

Data were analyzed using TagTrack v.1.8 (GyroTools Ltd, Zurich, Switzerland). For DENSE the echo signal was shifted by 20.8% in k-space (corresponding to 8 mm tag line distance), to create a phase image for HARP processing. CSPAMM data were processed directly with the HARP method using peak combination, demodulated peak combination and conventional single peak method [7].

The mid-contour inside the left ventricular myocardium was tracked starting from an end-diastolic frame. Initial contours were identical for DENSE and CSPAMM data to reduce observer variability. The left ventricle was segmented into six equidistant sectors and resulting curves for circumferential length and rotation were fitted by a fourth order polynomial. Comparison of both methods was performed using a Bland Altman test.

## Results

**Figure 1:**
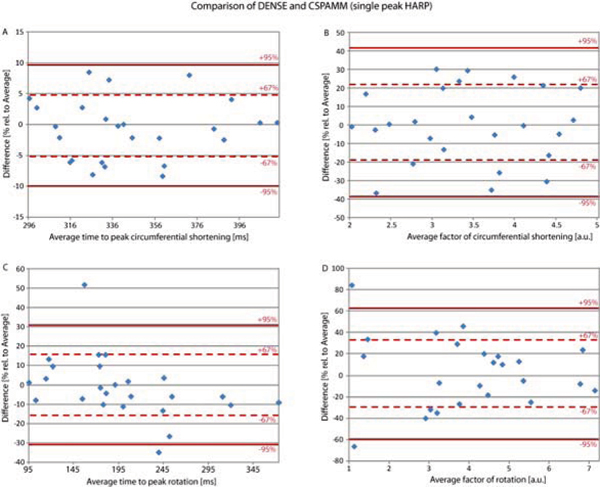
**Bland Altman tests for the comparison of DENSE and single peak HARP analysis are shown for time to peak circ**. shortening (A), relative circ. shortening (B), time to peak rotation (C) and amount of rotation (D).

Figure 1 shows Bland Altman plots for the comparison of DENSE and single peak HARP analysis. The difference between two measurements is presented for the time to peak circumferential shortening (A), the relative circumferential shortening (B), the time to peak rotation (C) and the amount of rotation (D), relative to the average of both measurements. Relative motion measurements show a bigger variation than timing measurements. Table 1 gives a detailed overview of the levels of agreement, bias and the first standard deviation of a comparison between DENSE and peak combined HARP, demodulated peak combined HARP and single peak HARP.

**Table 1:** The levels of agreement, bias and standard deviations from Bland Altman tests for the cardiac motion analysis are shown

	time to peak circ. shortening [% of average]				time to peak rotation [% of average]			
DENSE vs.	95%	-95%	bias	stdev	95%	-95%	bias	stdev
peak combination HARP	18.65	-22.84	-2.10	10.58	42.15	-47.36	-2.60	22.83
demodulated peak combination HARP	9.75	-9.16	0.30	4.82	31.10	-33.69	-1.30	16.53
single peak HARP	9.63	-9.99	-0.18	5.00	30.89	-30.91	-0.01	15.77
	**circ. shortening [% of average]**				**rotation [% of average]**			
DENSE vs.	95%	-95%	bias	stdev	95%	-95%	bias	stdev
peak combination HARP	45.18	-47.23	-1.03	23.57	50.44	-77.07	-13.31	32.53
demodulated peak combination HARP	35.29	-44.54	-4.62	20.36	84.44	-110.45	-13.00	49.72
single peak HARP	41.73	-38.81	1.46	20.55	62.82	-59.60	1.61	31.23

## Discussion

This work has presented a direct comparison of CSPAMM/HARP and DENSE. Results indicate that the both methods agree well when considering bias. However, considerable variation in individual values has been found which may partly be attributed to differences in breath hold position for the two different scans and phase unwrapping errors in demodulation of peak combination HARP. Future work is necessary to identify the cause of this variability.
